# *In vitro* Evaluation of Stainless Steel Crowns cemented with Resin-modified Glass Ionomer and Two New Self-adhesive Resin Cements

**DOI:** 10.5005/jp-journals-10005-1363

**Published:** 2016-09-27

**Authors:** Sidhant Pathak, KK Shashibhushan, P Poornima, VV Subba Reddy

**Affiliations:** 1 Postgraduate Student, Department of Pedodontics and Preventive Dentistry, College of Dental Sciences, Davangere, Karnataka, India; 2 Professor, Department of Pedodontics and Preventive Dentistry, College of Dental Sciences, Davangere, Karnataka, India; 3 Professor and Head, Department of Pedodontics and Preventive Dentistry, College of Dental Sciences, Davangere, Karnataka, India; 4 Professor, Department of Pedodontics and Preventive Dentistry, College of Dental Sciences, Davangere, Karnataka, India

**Keywords:** Resin-modified glass ionomer cement, Retentive strength, Self-adhesive resin cement, Stainless steel crown.

## Abstract

**Aims:**

To assess and compare the retentive strength of two dual-polymerized self-adhesive resin cements (RelyX U200, 3M ESPE & SmartCem2, Dentsply Caulk) and a resin-modified glass ionomer cement (RMGIC; RelyX Luting 2, 3M ESPE) on stainless steel crown (SSC).

**Materials and methods:**

Thirty extracted teeth were mounted on cold cured acrylic resin blocks exposing the crown till the cemento-enamel junction. Pretrimmed, precontoured SSC was selected for a particular tooth. Standardized tooth preparation for SSC was performed by single operator. The crowns were then luted with either RelyX U200 or SmartCem2 or RelyX Luting 2 cement. Retentive strength was tested using Instron universal testing machine. The retentive strength values were recorded and calculated by the formula: Load/Area.

**Statistical analysis:**

One-way analysis of variance was used for multiple comparisons followed by *post hoc* Tukey’s test for groupwise comparisons. Unpaired t-test was used for intergroup comparisons.

**Results:**

RelyX U200 showed significantly higher retentive strength than rest of the two cements (p < 0.001). No significant difference was found between the retentive strength of SmartCem2 and RelyX Luting 2 (p > 0.05).

**Conclusion:**

The retentive strength of dual-polymerized self-adhesive resin cements was better than RMGIC, and RelyX U200 significantly improved crown retention when compared with SmartCem2 and RelyX Luting 2.

**How to cite this article:**

Pathak S, Shashibhushan KK, Poornima P, Reddy VVS. *In vitro* Evaluation of Stainless Steel Crowns cemented with Resin-modified Glass Ionomer and Two New Self-adhesive Resin Cements. Int J Clin Pediatr Dent 2016;9(3):197-200.

## INTRODUCTION

One of the most common reasons for early loss of primary teeth is excessive tooth decay. Since introduced in 1950s by Dr. Humphrey, stainless steel crowns (SSCs) are unique coronal restorative materials that restore primary molars, which are grossly broken down.^1^Although SSCs have a high success rate, a key reason for its clinical failure is loss of crown due to cementation failure.^2,3^

The luting cement increases the retention of the restoration to the tooth preparation. The cement provides mechanical resistance to displacement of restoration and also resists fracture when load is applied to the restoration. The retention is further improved when the luting cement adheres to the tooth surface and restoration.^4^

Conventional glass ionomers are popular principally because they release fluoride that prevents recurrent caries.^5^ Glass ionomer cements (GICs), however, have prolonged maturity period and water sensitivity during the early setting reaction.^1^ Therefore, these cements are more susceptible to hydrolytic degradation than insoluble resin composite luting materials.^6^ The development of resin-modified glass ionomer cement (RMGIC) offers the benefit of both resins and conventional GIC, i.e., adhesion and fluoride release, along with improved physical properties that reduce the chance of cohesive failure.^7^ Currently, most resin cements use an etch and rinse or a self-etch adhesive in combination with a low viscosity dual-polymerizing resin cement.^8^ However, this multiple step bonding procedure is complex, technique-sensitive, and involves significant chair time. A new generation of self-adhesive resin cements has been developed recently that eliminates the need for etching, priming, and bonding as separate steps. These self-adhesive resin cements are based on new monomer, filler, and initiator formulations. The acidic monomer replaces the previous three steps by combining the use of adhesive and cement into a single application. These multifunctional phosphate-based acidic methacrylates can react with the basic fillers in the luting cement and the hydroxyapatite of the hard tooth tissue.^9^ Self-adhesive resin cements combine the high strength and low solubility advantages of resin cements with the characteristic ease of use of self-adhesive systems, making them highly attractive to the clinician.^10^

Hence, this study was undertaken to assess and compare the retentive strength of two dual-polymerized self-adhesive resin cement (RelyX U200, 3M ESPE & SmartCem2, Dentsply Caulk) and a RMGIC (RelyX Luting 2, 3M ESPE) on SSC.

## MATERIALS AND METHODS

Thirty extracted teeth were mounted on cold cured acrylic resin blocks exposing the crown till the cementoenamel junction. The appropriate crown for a particular tooth was selected by a trial and error procedure with respect to mesiodistal width and cervico-occlusal height of each tooth. Pretrimmed, precontoured SSCs were selected. Conventional tooth preparation for SSCs was performed by single operator. The occlusal surfaces of all teeth were reduced uniformly by using a straight fissure bur (#56). This was established by placing depth orientation grooves at the cuspal heights. The proximal surfaces were prepared with a tapered fissure bur (#848L) by removing all mesial and distal undercuts without leaving any ledges. All sharp line angles were rounded. For each prepared tooth, a prefabricated SSC was selected, fitted, contoured, and crimpled with contouring and crimping pliers. The crowns were removed and weldable wire hook was welded on occlusal surface of all crowns to facilitate an attachment for the universal testing machine. Specimens were divided into following three groups:

*Group I:* Rely X U200 group

*Group II:* Smart Cem2 group

*Group III:* Rely X Luting 2 group

**Fig. 1:**
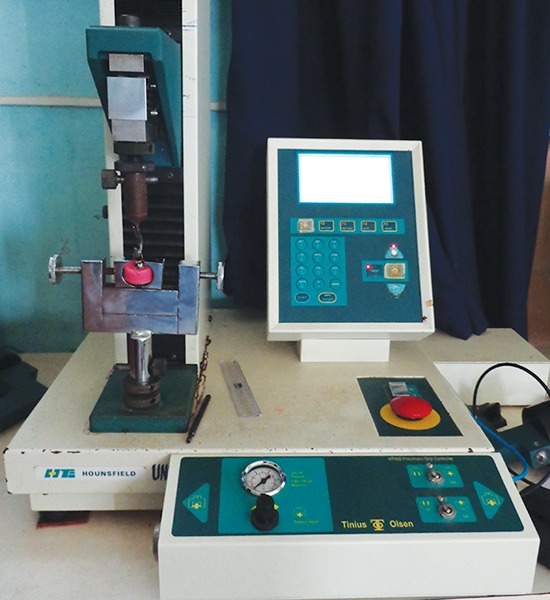
Specimen undergoing retentive strength testing in Instron universal testing machine

All teeth were cleaned with pumice and water before cementation. The crowns were luted with either RelyX U200 or Smart Cem2 or Rely X Luting 2 cements. All cements were used according to the manufacturer’s instructions at room temperature. They were then loaded into the crown and each crown was seated with finger pressure. After initial set, excess cement was removed from the crown tooth interface using an explorer. Artificial saliva was prepared by mixing 0.220 gm of calcium chloride, 1.07 gm of sodium phosphate, 1.68 gm of sodium bicarbonate, and 2 gm of sodium azide and then adding 1 L of distilled water. pH of saliva was adjusted to 7 to 7.4 using pH meter. The teeth were then stored in artificial saliva and incubated at 37°C for 24 hours. Retentive strength was tested using Instron universal testing machine. The machine was fitted with an Instron recorder. After stabilization of the specimen on the machine, load was applied, which gradually increased from zero reading to a point until the cemented crowns showed dislodgement and the corresponding value was noted from the testing machine computer monitor (Fig. 1). The same procedure was followed for all the specimens. The applied load was directly parallel to the long axis of the tooth during crown removal with a cross head speed of machine 0.05”/minute. The retentive strength values were recorded, expressed in terms of kgF/cm^2^ which was calculated by the formula:

Retentive strength = Load/Area

The surface areas of crowns were determined by cut opening of the crowns and their surfaces were developed on graph sheet and the areas of these developed surfaces were determined by counting the squares on the developed areas.

## RESULTS

*Group I:* Rely X U200 group: Mean retentive strength was 7.77 whereas highest value was 10.01 while lowest value was 5.64.

*Group II:* Smart Cem2 group: Mean retentive strength was 3.63 whereas highest value was 4.20 while lowest value was 2.51.

*Group III:* Rely X Luting 2 group: Mean retentive strength was 2.82 whereas highest value was 2.05 while lowest value was 3.53.

**Graph 1:**
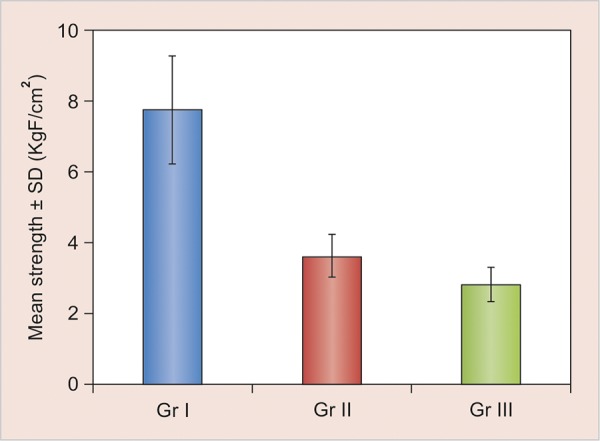
Intergroup comparison of mean retentive strength between groups I, II, and III

Comparison of luting cements showed retentive strength of Rely X U200 cement significantly higher than rest of the two cements (p < 0.001). No significant difference was found between the retentive strength of SmartCem 2 cement and Rely X Luting 2 cement (p > 0.05) (Table 1).Intergroup comparison of mean retentive strength between groups I, II, and III is also depicted graphically (Graph 1).

## DISCUSSION

Preformed crowns, most commonly known as the stainless steel crowns, began as a fairly crude metal tube closed on one side with a prestamped facsimile of a molar occlusal surface. A dentist required a significant amount of time and skill to festoon, crimp, and harden the margins to custom fit the tooth. Several iterations by manufacturers give today’s SSC a more realistic crown form with margins that are pretrimmed, prefestooned, and precrimped. Today’s crowns are much easier to place and often require minimal modifications from its manufactured form. The primary teeth are a temporary dentition with known life expectancies. By matching the right restoration with the expected life span of the tooth, the dental practitioner can succeed in providing a permanent restoration that will never have to be replaced. Stainless steel crown’s features of durability and full coverage for relatively low cost are not provided by other restorative materials, and the decision to place an SSC is best practice.^11^ Primary molars were selected for this study because SSCs are more widely used on primary molars to prevent premature tooth loss and development of future malocclusion. Pretrimmed and precontoured SSCs were used in this study because to standardize the surface area of the crowns as in case of other type of crowns trimming is necessary which gives an intra-clinician variation in surface area and the specimens were stored in the artificial saliva because it simulates human saliva. There are several factors that have an influence on the retention of a fixed prosthesis. Generally, greater forces are required to dislodge the crown cemented with a material that has higher tensile strength. Undoubtedly, other properties, such as compressive strength, shear strength, fracture toughness, and film thickness are also involved. The use of cement with potential chemical bonding to the tooth and prosthetic surface may also be used to enhance retention.^12^ Though conventional glass ionomer interacts interfacially with the tooth structure creating covalent bonds, the role of these bonds is not significant in increasing retention.^13^ The low retention of GIC could be due to spontaneous cohesive fracture of the cement, due to high stress generated by contraction on setting, compounded by constraints of cement adhesion to the crown and dentin walls, in geometric configuration where few opportunities for relief of stress by plastic deformation or cement flow exists. The lower tensile strength and fracture toughness of conventional GIC is another cause of fracture at lower loads.^14^ Both conventional GIC and RMGIC dehydrate and contract extremely rapidly in air or humidity. Addition of resin to the brittle composition of conventional GIC significantly increases its fracture toughness. The compressive strength, flexural strength, and modulus of elasticity of resin composites are significantly higher than conventional GICs and RMGICs.^4^ A recent report demonstrated that RMGIC acid-base and visible light polymerization reactions inhibit one another during the early phases of setting, which may explain the low retentive strength of RMGIC.^15^ Resin cements typically exhibit higher retentive strengths when light activated due to the higher degree of conversion under light polymerization conditions.^10^ Presence of phosphate esters in the primer would decalcify dentin or enamel, thus improving the micromechanical bonding between tooth’s hard tissue and resin cement. Ionic bonding between the negatively charged phosphate ester monomer and the positively charged calcium ions on tooth may occur.^1^

**Table 1:** **Table 1:** Intergroup comparison of mean retentive strength between groups I, II, and III

*Groups*				*Mean strength ± SD* *(kgF/CM^2^)*	
I				7.77 ± 1.53	
II				3.63 ± 0.60	
III				2.82 ± 0.50	
ANOVA		F		71.35	
		P		0.00*	
One-way ANOVA					
Groupwise comparisons					
*Groups compared*		*Mean diff*		*p-value*	
I *vs* II		4.14		0.00*	
I *vs* III		4.95		0.00*	
II *vs* III		0.81		0.18, NS	

*Post hoc* Tukey’s test; *p < 0.001, hs; p > 0.05, NS: Nonsignificant

## CONCLUSION

Within the limitations of the present *in vitro* study, the following conclusions may be drawn:

- The retentive strength of dual-polymerized self-adhesive resin cements was better than RMGIC.
- RelyX U200 significantly improved crown retention when compared with SmartCem2 and RelyX Luting 2.
- SmartCem 2 showed better retentive strength than RelyX Luting 2, but this difference was not significant.
- Regardless of the utmost care taken in conducting this study efficiently, further research is still necessary in this direction.
